# Holistic Education for a Resilient Future: An Integrated Biomimetic Approach for Architectural Pedagogy

**DOI:** 10.3390/biomimetics10060369

**Published:** 2025-06-05

**Authors:** Lidia Badarnah

**Affiliations:** School of Architecture and Environment, College of Arts, Technology and Environment, University of the West of England (UWE), Bristol BS16 1QY, UK; lidia.badarnah@uwe.ac.uk

**Keywords:** inclusivity, creativity, biomimicry, social, adaptive, building envelope, ecology, problem-solving, experiential, constructivist

## Abstract

The pressing need to address climate change and environmentally related challenges highlights the importance of reimagining educational approaches to equip students with the skills required for innovation and sustainability. This study proposes a novel holistic pedagogic framework for architectural education that integrates biomimicry, systems thinking, and Bloom’s Revised Taxonomy to advance innovation, sustainability, and transformative learning. Developed through a triangulated methodological approach—combining reflective practitioner inquiry, design-based research, and conceptual model development—the framework draws from multiple theoretical perspectives to create a cognitively structured, interdisciplinary, and ecologically grounded educational model. Bloom’s Taxonomy provides a scaffold for learning progression, while the Function–Structure–Behavior (FSB) schema enhances the establishment of cross-disciplinary bridges to enable students to address complex design challenges. The framework is informed by insights from the literature and patterns observed in bio-inspired studios, student projects, and interdisciplinary workshops. These examples highlight how the approach supports systems thinking, ecological literacy, and ethical decision-making through iterative, experiential, and metacognitive learning. Rather than offering a fixed intervention, the framework is presented as a flexible, adaptable model that aligns learning outcomes with real-world complexity. It enables learners to navigate interdisciplinary knowledge, reflect critically on design processes and co-create regenerative solutions. By positioning nature as *mentor*, *model*, and *measure*, this pedagogic framework reimagines architectural education as a catalyst for sustainability and systemic change in the built environment.

## 1. Introduction

The increasing urgency to address climate change, resource depletion, and environmental degradation necessitates a fundamental transformation in architectural educational approaches, particularly in preparing future designers to tackle complex environmental and societal challenges. Transformative learning requires both students and educators to engage in critical reflection, interdisciplinary collaboration, and participatory pedagogies [1]. As Leal Filho et al. [2] argue, sustainability education must move beyond content delivery to foster ethical, cultural, and systemic change within higher education institutions. This calls for a whole-university approach, where teaching, research, operations, and community engagement are aligned to promote sustainability. By empowering faculty to redesign curricula and encouraging students to engage with real-world, cross-disciplinary challenges, institutions can create learning environments that cultivate systems thinking, innovation, and sustainability competencies.

Architecture plays a pivotal role in shaping the built environment, directly influencing energy consumption, material use, and ecological resilience. The built environment accounts for nearly 40% of global energy consumption and is a major contributor to carbon emissions [3]. Design decisions—from material selection and passive/active climate strategies to urban form and land use—have lasting impacts on environmental performance and human well-being [4]. Despite this, traditional architectural education often treats environmental, technical, and design considerations as isolated fields, limiting students’ ability to synthesize across domains or anticipate long-term impacts [5]. There is a pressing need to reframe architectural pedagogy to equip learners with the interdisciplinary and systemic competencies necessary for sustainable design. In the context of sustainability, education must move beyond traditional linear approaches to encompass the complexity and uncertainty of real-world systems. Posch and Steiner [6] argue that integrating research and teaching fosters innovation by engaging learners in processes that begin with acquiring knowledge, advance through deeper understanding, and culminate in the application of that knowledge in diverse and dynamic contexts.

Biomimetics—the practice of learning from and emulating nature’s strategies—offers a transformative opportunity in education [7]. Grounded in systems thinking, biomimicry fosters creativity, ecological literacy, and interdisciplinary problem-solving, making it a compelling framework for equipping students to address real-world sustainability challenges [5,8,9]. Natural systems are inherently resilient, resource-efficient, and interconnected; applying these principles to design encourages a shift from human-centered to ecosystem-centered thinking [10,11,12]. As such, biomimicry offers not only technical inspiration but also ethical and epistemological foundations for regenerative design, aligning architecture with long-term environmental stewardship and resilience.

To fully respond to the complexity of sustainability challenges, education must embrace integrative, adaptive, and experiential learning. This requires moving beyond traditional pedagogies and developing curricula that engage students in iterative problem-solving, critical reflection, and collaboration across disciplines [13]. In this context, biomimicry acts as a “superdiscipline” [14], linking biology, design, and engineering to develop systemic solutions through experiential and constructivist approaches.

Building on Ison’s [15] work on systems thinking in higher education, this study positions systems thinking not merely as a pedagogical strategy, but as a foundational paradigm for reimagining education itself. Ison [15] contends that meaningful transformation requires second-order change—rethinking of values, relationships, and system dynamics—rather than the superficial reform of existing structures. Biomimetic education exemplifies this shift by promoting holistic, interconnected learning aligned with the complexity of socio-ecological systems. In doing so, it reframes education as a dynamic, communicative, and emergent process.

Wanieck et al. [16] further emphasize that effective biomimicry education demands methodological clarity, tool-based training, and tailored curricula that address the diverse learning needs of students across disciplines. Such pedagogies must cultivate cognitive flexibility, interdisciplinary competence, and creative problem-solving—capabilities essential for navigating sustainability transitions. An integrated approach to education for sustainability and innovation emphasizes project-based learning (PBL), experiential engagement, and systemic inquiry [17,18]. The recognition of the Anthropocene—a new epoch shaped by human-induced planetary change—requires a pedagogical reorientation. Traditional models prioritizing content delivery and efficiency fail to support the adaptive, systems-oriented thinking required to respond to global crises such as climate change, biodiversity loss, and resource scarcity [19].

Biomimicry provides a promising foundation for this reorientation, offering both conceptual depth and practical guidance through its emphasis on nature as *model*, *measure*, and *mentor* [20]. By drawing on 3.8 billion years of evolutionary solutions, biomimicry fosters design thinking rooted in adaptability, resilience, and multifunctionality [21]. In education, it facilitates integrative learning environments where students engage in knowledge construction, real-world experimentation, and reflective practice [22].

Educational systems, like ecosystems, thrive on diversity, feedback, and adaptation. Just as natural systems evolve through interaction and interdependence, learning environments must evolve to prepare students for uncertain, rapidly changing futures. As Weinstock [23] notes, learning environments inspired by nature can promote adaptability, collaboration, and responsiveness—core qualities for both sustainable design and transformative education.

This paper explores the integration of systems thinking and biomimetic design into architectural pedagogy. It proposes a holistic educational framework that emphasizes experiential learning, interdisciplinary collaboration, and curriculum integration. Grounded in cognitive theory and informed by natural systems, the framework is designed to equip students with the knowledge, values, and competencies necessary to innovate and thrive in a complex, sustainability-oriented world. Through this approach, the paper contributes to the discourse on pedagogic transformation, offering a model for reimagining architectural education as a catalyst for regenerative futures.

## 2. Methodology

This study is grounded in the pedagogical understanding that learning is inherently situated, deep, and developmental, as articulated by Budwig [24]. That is, learning is situated in the social, cultural, and disciplinary context in which it occurs; deep in that it engages learners intellectually through critical reflection, meaning-making, and conceptual integration; and developmental in that it builds progressively through cognitive processes over time. These dimensions are particularly relevant for designing educational frameworks that integrate biomimicry and systems thinking—domains that require learners to navigate real-world complexity through interdisciplinary collaboration and reflective engagement.

Accordingly, the methodology adopted in this study combines reflective practitioner inquiry, design-based research, and conceptual model development to support both pedagogical evolution and the creation of a transformative learning environment. A theoretical triangulation approach [25] underpins the framework’s development, drawing on intersecting perspectives from cognitive learning theory, biomimetic design, systems thinking, and constructivist pedagogy. This triangulated lens ensures a robust, multidimensional foundation for the proposed model, supporting its adaptability across various educational contexts.

The framework is built on two central pillars: Bloom’s Revised Taxonomy [26], used as a cognitive scaffold, and biomimicry [20], employed as a transdisciplinary problem-solving methodology. Bloom’s Taxonomy provided a structured progression for mapping learning outcomes, pedagogical strategies, student roles, and competencies—from foundational knowledge to higher-order problem-solving and creative synthesis. This mapping is outlined in Table 1, which aligns cognitive levels with the corresponding domains of learning. Biomimicry complements this structure by offering a methodology for exploring nature’s strategies and abstracting them into architectural solutions, thereby encouraging interdisciplinary integration, systems thinking, and sustainability literacy. The framework was further developed through conceptual modeling techniques [27], using mapping tools such as matrices and radial diagrams to visualize interrelationships among cognitive levels, pedagogic approaches, subject domains, and learning environments. This visualization process supports coherence across dimensions and strengthens the framework’s applicability for curriculum design.

A key addition to the holistic framework is the integration of the Function–Structure–Behavior (FSB) model, which is often used in biomimicry and systems thinking [28,29]. This triadic model facilitates the establishment of cross-disciplinary bridges by offering a shared analytical lens through which students and educators from fields such as biology, architecture, and engineering can engage in meaningful dialogue. It enables learners to analyze biological systems by distinguishing their purpose (function), organization (structure), and dynamic interactions or transformations (behavior). Embedding this model enhances students’ ability to interpret and apply complex ecological strategies within architectural design challenges, while supporting the alignment of pedagogic goals with systems-oriented learning. It also strengthens the translatability between scientific understanding and creative design, enabling interdisciplinary teams to co-develop solutions with conceptual clarity and shared language.

**Figure 1:**
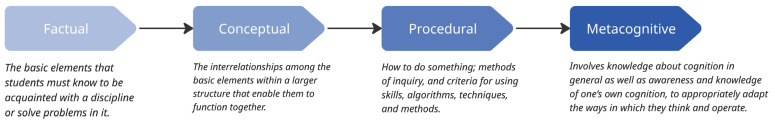
Knowledge dimensions of Bloom’s Revised Taxonomy [26]. Source: Figure by author.

Importantly, the framework emphasizes ‘metacognition’ as a driver of educational transformation. As illustrated in Figure 1, metacognitive processes—such as questioning assumptions, evaluating strategies, and regulating one’s learning—are vital in sustainability education, where students must navigate ambiguity, feedback loops, and interconnected systems [30,31]. Within a biomimetic context, this entails reflecting not only on what strategies are borrowed from nature, but also how and why these strategies are abstracted and applied. By moving between disciplinary boundaries, collaborative environments, and iterative design processes, learners cultivate adaptability, ethical awareness, and system-level reasoning.

In summary, this methodology offers a multi-layered, triangulated approach to framework development, integrating theoretical, experiential, and visual tools. It ensures that the proposed pedagogic model supports deep, transformative learning and equips students with the competencies needed to engage critically and creatively with the challenges of sustainability in architectural education.

## 3. Biomimetic Design Methods

**Figure 2:**
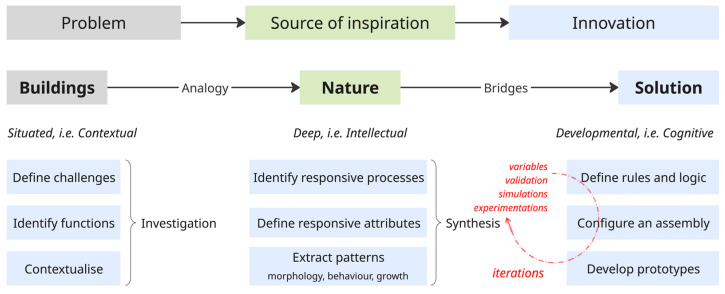
Biomimetic design process across domains aligned with Budwig’s understanding of learning—i.e., situated, deep, and developmental. Source: Figure by author.

Given the interdisciplinary nature of biomimetics, several methods have been developed to facilitate its application in the design process, each emphasizing different strategies and stages of innovation [32,33,34]. These include natural language processing [35], to analogical reasoning [36], structure-behavior-function schema [37] and creativity-driven tools such as BioTRIZ [38]. Collectively, methods employing analogical reasoning, function-structure mapping, and pattern recognition offer powerful tools for fostering creativity, interdisciplinary synthesis, and innovative problem-solving capacities [39]. The methods typically follow two main approaches: problem-based (bottom-up) and solution-based (top-down). In architectural design, the problem-based approach is particularly prominent, beginning with a design challenge and seeking relevant biological models that can inform a solution. However, this approach presents two key challenges: the breadth of possible biological analogs and the difficulty of analyzing and abstracting relevant functions and principles across domains [40]. To facilitate transitions between biology and design, the dimensions of function (situated and context-specific) and pattern (deep and transferable) have been identified as essential [10], enabling iterative synthesis and evaluation of design concepts—an approach visually represented in Figure 2.

Pedagogical practices increasingly reflect a shift away from technocentric models of sustainability toward ethically grounded and ecologically integrated frameworks. McCormick and Thaddeus [41] propose a biomimetic epistemology that challenges conventional architectural paradigms by embedding environmental philosophy into studio-based learning. Their work advocates for design processes that move beyond form imitation to engage with ecological principles at a deeper, systems level—embracing impermanence, interdependence, and resilience. For example, in their studio at UNC Charlotte, students abstracted biological strategies from marine species and applied them to projects addressing climate-induced displacement, culminating in design concepts that integrate cultural memory with environmental responsiveness. This approach illustrates how biomimetic education can cultivate ethical awareness and ecological literacy, promoting transformative learning that aligns with the complexities of the Anthropocene.

Well-structured visuals and conceptual tools—such as exploration models, analyzing matrices, concept maps, and abstraction frameworks—enable designers and students to identify relationships, synthesize information, and navigate the interconnected aspects of natural and engineered systems [42,43,44]. These tools support interdisciplinary collaboration by facilitating clarity and coherence, as well as by providing language bridges for biologists, engineers, and designers. Effective data representation also plays a key role in systematically organizing and integrating complex information, enhancing understanding and creativity while supporting decision-making in the biomimetic design process [33].

**Figure 3:**
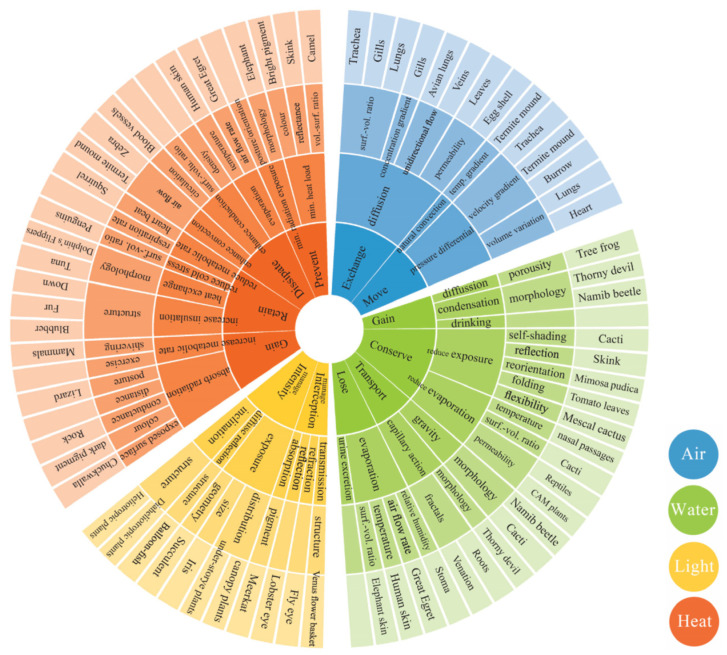
Multi-regulation exploration model for air, water, light, and heat strategies from nature, adapted from [40]. Inner band represents key functions, next band branching out from functions represents processes, the following band branching out from processes represents factors, then most outer band represents the relevant models from nature. This cluster has the capacity to grow with new inputs.

In architectural contexts, most biomimetic solutions tend to focus on a single environmental aspect at a time. However, a building envelope, for instance, must simultaneously manage multiple interrelated environmental factors, such as air, heat, water, and light. These aspects are inherently interconnected, and the regulation of one often depends on the regulation of another. As part of the BioGen methodology [33], comprehensive biological investigations have guided the development of exploration models, and their combination has led to the creation of a function-based multi-regulation model designed to address complex and multifaceted problems, as presented in Figure 3. These models play a pivotal role in systematically organizing knowledge and enabling relevant data retrieval to support cross-disciplinary integration [40].

By adopting biomimetic approaches and drawing inspiration from nature’s principles, these methods equip learners with the ability to analyze complex systems and translate biological strategies into innovative solutions [45,46,47,48]. However, their full educational potential lies in how they contribute to a broader, holistic pedagogic framework—one that integrates biomimetics not as an isolated method, but as a key driver for cultivating innovation, sustainability, and systems thinking. Grounded in constructivist and experiential learning, this framework supports the development of not only technical competencies, but also the values, mindsets, and capacities necessary to navigate and address interconnected global challenges. The following sections examine how these foundational principles are embedded in education, culminating in a holistic educational model that connects theory, practice, and purpose through the lens of biomimetic thinking.

## 4. Sustainability and Innovation in Education

Sustainability in education has become increasingly essential in equipping learners with the knowledge, skills, and values needed to address complex environmental and societal challenges, emphasizing transformative, action-oriented learning approaches that foster systems thinking, interdisciplinary collaboration, and long-term resilience [13]. At its core, sustainability education focuses on understanding the dynamic relationships between ecological, social, technological, and economic systems. It aims to instill the values of stewardship, equity, and long-term thinking while promoting actionable knowledge and ethical responsibility [49]. By teaching students to assess the environmental impacts of human activities and design solutions aligned with the principles of resource efficiency and regeneration, sustainability education can effectively bridge theory and practice. In doing so, it challenges learners to consider not only technical feasibility but also social and environmental justice [49].

**Figure 4:**
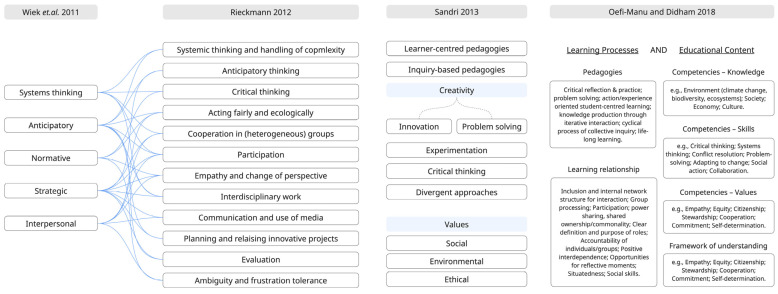
Representation of the required capabilities and competencies for sustainable development in higher education [51,52,53]. Source: Figure by author.

According to Wiek, et al. [50], five core competencies underpin sustainability education: systems thinking, anticipatory, normative, strategic, and interpersonal. Rieckmann [51], through empirical studies, expands this framework with twelve key competencies that higher education should foster. These include critical thinking, integrated problem-solving, and collaboration, which are crucial for addressing “wicked problems” in sustainability contexts. Sandri [52] adds on the significance of creativity for sustainable development, especially in addressing the complex, interconnected, and “wicked” problems that characterize our current global challenges. Sandri [52] emphasizes the critical role of creativity in sustainable development, arguing that such challenges require not only transmitting existing knowledge but also generating new, imaginative, and context-sensitive solutions. Despite its significance, creativity remains underrepresented in many sustainability education curricula. To empower students as future change agents, creativity must be embedded explicitly through learner-centered, inquiry-based pedagogies that support experimentation, critical and divergent thinking, and problem-solving. Embedding creativity explicitly in sustainability education not only challenges traditional models of knowledge delivery but also cultivates the adaptive and visionary thinking essential for transformative action. As Ofei-Manu and Didham [53] suggest, a quality education for sustainable development must integrate progressive pedagogies, cooperative learning, values-based content, and a systems worldview. This approach enables education to move beyond passive knowledge transfer, supporting critical thinking, agency, and practical skills for sustainable action. Figure 4 presents these interrelated considerations based on findings from [50,51,52], and from the integrated approach by Ofei-Manu and Didham [53].

Innovation in education, in this context, involves cultivating a mindset that values creativity, critical thinking, and a willingness to iterate, fail, and adapt—processes that mirror natural evolution. Designing environments for innovation, where learning and practice can flourish requires moving beyond conventional models of instruction toward more participatory and developmental approaches. As Budwig [24] notes, effective learning environments support guided emergence, where educators serve as facilitators of collaborative inquiry. These spaces foster shared purpose, enable formal and informal collaboration, and provide reflective tools that help students construct knowledge, co-create meaning and assume responsibility for their learning. These principles are especially relevant in sustainability and biomimetic education, where learners must address real-world complexity through ecological insight and interdisciplinary dialogue.

Despite the growing awareness of sustainability challenges, many universities face structural barriers to implementing innovation and sustainability effectively. Ávila et al. [54] highlight persistent challenges, including lack of institutional leadership and commitment, inadequate funding, and fragmented strategies. Rigid disciplinary boundaries, bureaucratic inertia, and insufficient stakeholder engagement further hinder efforts to integrate sustainability into curricula and research. The absence of accountability frameworks and institutional incentives compounds the problem, pointing to the need for systemic reform to support transformative learning environments.

**Figure 5:**
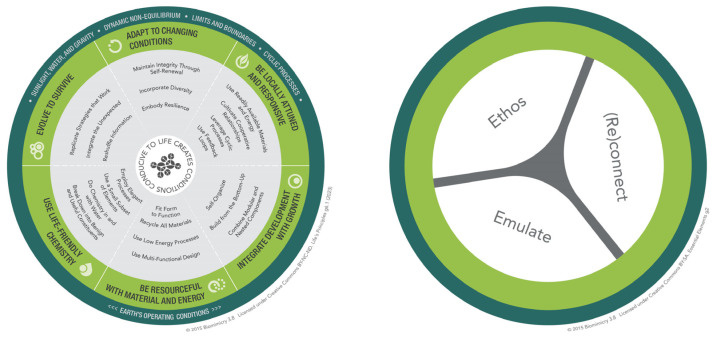
Left: Biomimicry life’s principles and their sub principles. Right: Essential elements. CC BY-NC-ND. Permission granted by Biomimicry 3.8 under Creative Commons.

Biomimicry offers a compelling lens through which to promote both sustainability and innovation in education [55]. It encourages students to see nature as a model, measure, and mentor [20], and to apply its strategies to solve complex problems creatively and responsibly. For example, the buoyancy and structural resilience of organisms like the Venus flower basket, giant kelp, and red mangrove provide design cues for flood-responsive architectural solutions [56]. Tools such as Life’s Principles (Figure 5) offer accessible, ecologically grounded guidelines—e.g., Adapt to Changing Conditions, Use Life-Friendly Chemistry—for sustainable design thinking [57,58]. Teaching students to use these principles supports regenerative design, aligning innovation with ethical and ecological values [21,59]. The systemic approaches of biomimicry, as outlined in established frameworks (e.g., [10,60]), enable learners to approach problems holistically, fostering the capacities needed for long-term adaptability and transformation. Importantly, this shift from conceptual understanding to applied innovation demands learning environments that support experimentation, synthesis, and feedback.

In this regard, the architectural design studio plays a central pedagogical role. It provides a dynamic, project-based learning environment where students engage in iterative design, interdisciplinary collaboration, and critical reflection [61,62]. Within the studio, students move beyond passive learning to actively construct and test ideas—integrating material experimentation, site analysis, and conceptual development [63]. When framed through a biomimetic lens, the studio becomes a space of inquiry-led discovery, where students abstract nature’s strategies and apply them to urgent ecological and social challenges [10,31]. Such studio settings support authentic, interdisciplinary learning, enabling students to navigate uncertainty, negotiate feedback, and synthesize knowledge across domains [64].

**Figure 6:**
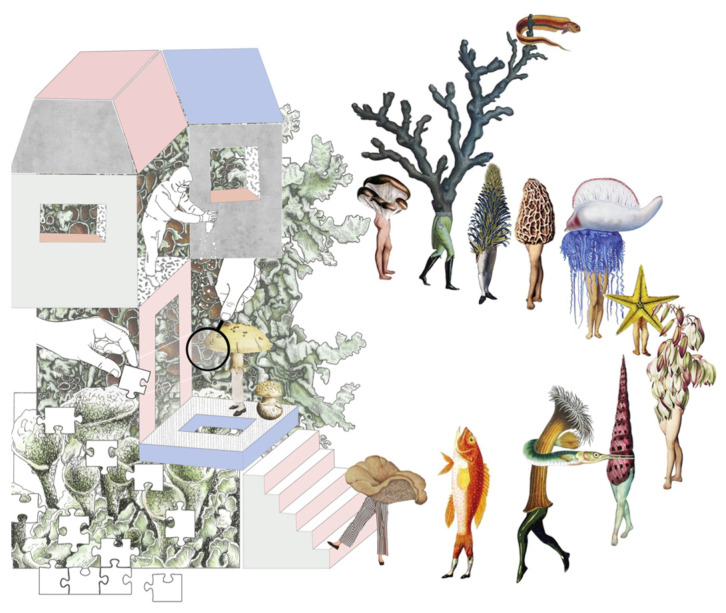
The vision of a collaborative campus proposal integrating biodiversity, sustainability, and interdisciplinary innovation. The Material Incubator fosters hands-on learning and experimentation with natural materials, emphasizing endemic design principles and ecological stewardship. The lens highlights the investigative nature of the proposal. Used with permission; image courtesy of Klaudia Kery 2022/23.

Developing design briefs that embed sustainability and environmental principles, especially through climatic responsiveness and purposeful challenges, is essential for fostering meaningful learning. When briefs address concrete environmental issues, they prompt students to integrate contextual variables—climate data, materials, and user needs—into their design reasoning [31,61,65]. Framing sustainability as a design driver rather than an afterthought encourages more intentional and innovative outcomes. When combined with biomimicry, these briefs prompt students to ask how nature solves similar problems, guiding them toward solutions that are efficient, adaptive, and regenerative [10,66]. For example, a student from Studio B (Emergent Biomes group, MArch, UWE Bristol) proposed the Material Incubator project (Figure 6) as a response to a brief rooted in specific environmental and social constraints. Located near a biodiverse national park, the project proposed a collaborative educational campus that transforms locally available materials—such as mushrooms, algae, and moss—into sustainable design elements. By integrating biology, materials science, and architecture, the proposal exemplifies endemic design, drawing on regional ecosystems and cultural narratives to generate context-specific, sustainable innovation. The project highlights how hands-on experimentation and interdisciplinary collaboration enable students to explore ecological principles while addressing real-world challenges.

In summary, innovation and sustainability in education thrive when grounded in ecological awareness, material sensitivity, and clearly defined design intent. However, for students to meaningfully engage with such complexity, they must develop the capacity to recognize patterns, feedback loops, and systemic interdependencies. This is where systems thinking becomes foundational—empowering learners to connect ideas across scales and disciplines, and equipping them to design for resilience, regeneration, and transformation.

## 5. Systems Thinking as a Foundation

Sustainability science increasingly calls for new forms of interaction that integrate the natural and social sciences while strengthening connections between academic research and societal needs [13]. In this context, evolving conceptions of science, knowledge, and practice highlight the need for transformative approaches to learning, teaching, and research. To foster systemic change, universities must meet several key requisites: integrating science with society, fostering real-world and participatory learning environments, embracing value pluralism, rethinking assessment practices, and cultivating competencies like systems thinking and normative reasoning [13]. Transformative and transgressive social learning approaches respond to the limitations of conventional higher education by enabling multi-actor engagement, promoting cognitive justice, and building systemic agency. These approaches support learners in disrupting unsustainable paradigms and co-creating more just and sustainable futures [1].

At the heart of these pedagogical transformations lies systems thinking, a foundational lens in holistic education. It emphasizes the interconnectedness and interdependence of systems, encouraging learners to understand problems as part of a larger, dynamic whole [67]. In doing so, it promotes the analysis of feedback loops, emergent properties, and pattern recognition—capacities essential for addressing multifaceted challenges such as climate change, urban resilience, and global sustainability, where solutions demand a balance of ecological, social, and technological considerations [68,69]. Systems thinking shifts the focus from linear cause-and-effect reasoning to an integrative perspective, helping students synthesize knowledge across disciplines and apply it adaptively and innovatively [5,8].

**Figure 7:**
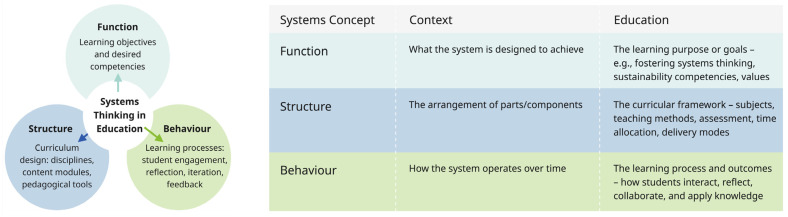
Key components of systems thinking. (**Left**) Interrelated representation of function, structure and behavior. (**Right**) Relating function, structure, and behavior to education.

Applying systems thinking in education also enables a shift in how learning environments are conceptualized: as dynamic systems themselves, composed of interrelated elements serving purposeful outcomes. Drawing from systems theory, every system can be understood through three interrelated dimensions: function (what it aims to achieve), structure (how its components are organized), and behavior (how it evolves over time and responds to change) [8,70]. In education, function may refer to learning goals such as developing sustainability competencies; structure corresponds to curricular and institutional design, including the integration of disciplines, pedagogical strategies, and institutional frameworks; and behavior reflects how students learn, adapt, and engage with their environments. Figure 7 illustrates this triadic model. Designing educational experiences through this lens enables more coherent, regenerative, and responsive curricula—particularly relevant in sustainability and biomimicry, where systems-level understanding is crucial [50,71].

Traditional reforms often fall short by addressing isolated components without considering the dynamic interactions across an education system [72]. In contrast, a systems approach emphasizes the need for coherent, cross-sectoral change driven by feedback-informed leadership, inclusive coalitions, and iterative adaptation. Ndaruhutse, Jones, and Riggall [72] outline how countries like Finland have restructured their education systems to function as adaptive wholes: aligning learner development with decentralized structures, trusting teacher professionalism, and supporting systemic behaviors such as collaboration, formative assessment, and local innovation. This alignment enabled Finland to achieve equitable, high-quality outcomes while maintaining resilience to changing societal needs.

York, Lavi, Dori, and Orgill [29] demonstrate how systems thinking can be effectively integrated into STEM curricula by encouraging students to view problems through the function–structure–behavior lens. For example, in urban water systems units, students examine infrastructure design (structure), understand its role in managing runoff (function), and model real-time flow patterns and pollutant dynamics (behavior). Similarly, in ecosystem or energy system modules, students map biotic interactions, analyze feedback loops, and predict performance under changing conditions. These exercises build integrative reasoning skills and prepare learners to identify leverage points for sustainable change.

This triadic lens of function-structure-behavior is especially powerful in biomimetic education, which inherently draws on the analysis and emulation of natural systems. For instance, in designing a building inspired by termite mounds, students examine the function of thermoregulation, study the structure of ventilation channels, and simulate behavioral responses to airflow and temperature changes over time. Such design processes require an understanding of how biological systems maintain equilibrium through interdependencies—insights that can be applied to achieve energy efficiency and resilience in architecture [73]. Likewise, in urban design contexts, students analyze green infrastructure like bioswales or green roofs to explore how structure and behavior intersect to regulate water, mitigate heat, and support biodiversity [74].

Yet, systems thinking is not automatically acquired—it must be cultivated through intentional instructional design. As Elsawah et al. [75] note, teaching systems thinking effectively requires a structured integration of systems concepts, modeling tools, and applied case-based learning. Their “three-thread” approach—comprising systems concepts, modeling tools, and applied case-based learning—demonstrates how scaffolded exposure to complexity, interdependencies, and feedback strengthens students’ capacity for systems reasoning. This aligns closely with biomimetic pedagogy, where learning occurs through iterative investigation of function–structure–behavior relationships across both natural and built systems.

To support these approaches, tools such as concept mapping, causal loop diagrams, and systems modeling can be used to externalize thinking and support interdisciplinary learning. Concept maps, for example, help students visualize complex interactions and relationships across ecological, social, and technical domains [76,77,78]. When applied to projects involving urban planning, renewable energy systems, or community design, such tools expose feedback loops, performance trade-offs, and opportunities for intervention [79]. This not only strengthens creative and analytical reasoning, but also builds ecological literacy and strategic thinking.

Ultimately, embedding the function–structure–behavior triad into education transforms systems thinking from a mindset into a pedagogical method. It prepares learners to reason across domains, respond to uncertainty, and design for resilience. However, cultivating these abilities requires immersive, inquiry-based environments that support exploration, reflection, and feedback—conditions best enabled by constructivist and experiential learning, which are explored in the following section.

## 6. Constructivist and Experiential Learning

Constructivist and experiential learning theories provide a dynamic foundation for biomimicry education, emphasizing active engagement, inquiry, and real-world application. These approaches align with biomimicry’s central focus on observing, identifying, analyzing, and applying nature’s principles to human challenges, thereby fostering meaningful connections between theory and practice.

According to Piaget [80] and Vygotsky [81] constructivist learning occurs when knowledge is actively built through the learner’s interaction with their environment and their existing understanding. Within a biomimetic context, students investigate natural systems, identify core principles such as adaptation and efficiency, and apply these insights to complex human problems. This requires a dynamic interplay between disciplines—moving from biological research to conceptual design and practical prototyping [82]. For example, by studying the microstructures that make lotus leaves hydrophobic, students can conceptualize and prototype self-cleaning surfaces for architectural applications. This iterative process fosters both critical thinking and interdisciplinary collaboration, strengthening problem-solving capacities.

Experiential learning, as conceptualized by Kolb [17], complements constructivism by emphasizing learning through direct experience, reflection, and adaptation. In biomimicry, this takes the form of “design experiments” or “design research,” where students transform observations into iterative prototypes. This aligns with Herbert Simon’s foundational distinction between the “natural sciences” and “design sciences” in The Sciences of the Artificial [83]. Simon argued that design required its own rigorous methodologies—an idea further developed through the emergence of design experiments in the 1990s [84,85,86]. These approaches help formalize how design knowledge is constructed and tested, particularly within education.

Recent studies have highlighted the availability and growing sophistication of biomimetic tools that support both constructivist and experiential learning. Zhang et al. [87] catalogued over 100 biomimetic tools—including BioCards, BioTRIZ, and the AI-powered BIDARA—organized across the design process from biological analysis to application and evaluation. These tools encourage students to explore structure–function–behavior relationships in nature and guide them through ideation and abstraction. In doing so, they support self-guided discovery, iteration, and collaborative learning. However, Zhang, Kestem, Wommer, and Wanieck [87] also identified a gap in tools that support solution-based and evaluative stages, reinforcing the need for integrated reflective practices and feedback loops in design education. Embedding these tools within studio-based curricula deepens systems thinking and strengthens students’ capacity to synthesize biology, design, and technology.

Beyond classroom tools, the physical and social learning environments play a critical role. Constructivist and experiential pedagogies emphasize place-based and community-embedded learning, where knowledge is co-produced through engagement with real-world problems. Ramasubramanian and Pincetl [88] highlight examples such as San José’s CommUniverCity and Portland’s Community Watershed Stewardship Program, which immerse students in situated, interdisciplinary sustainability challenges. These experiences require learners to grapple with uncertainty, integrate stakeholder perspectives, and reflect on their actions—mimicking the feedback-rich nature of ecological systems. Such environments cultivate civic responsibility, adaptive problem-solving, and systems thinking—essential skills for sustainability-oriented design.

**Figure 8:**
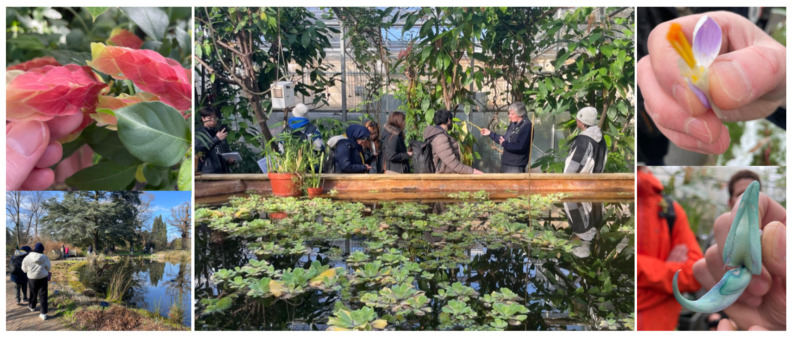
Pictures from the visit to the University of Bristol Botanic Garden (guided by curator Mr. Nick Wray) with the students of the ‘Crafting Systems’ module as an experiential learning activity, providing students with the opportunity to observe and analyze natural systems, explore biological principles, and draw inspiration for biomimetic design solutions. Source: Photos by author.

Biomimetics also benefits from rich interdisciplinary networks that connect students with campus fabrication labs, industry partners, and other academic departments. Plants, as one of the most commonly used biological analogues in biomimetic architectural design [89,90], have inspired numerous solar-responsive and water-harvesting innovations [44,91,92,93]. Structured visits to botanic gardens (as shown in Figure 8) can serve as key moments in the biomimetic design process—supporting early-stage abstraction and collaboration with biologists and environmental scientists.

**Figure 9:**
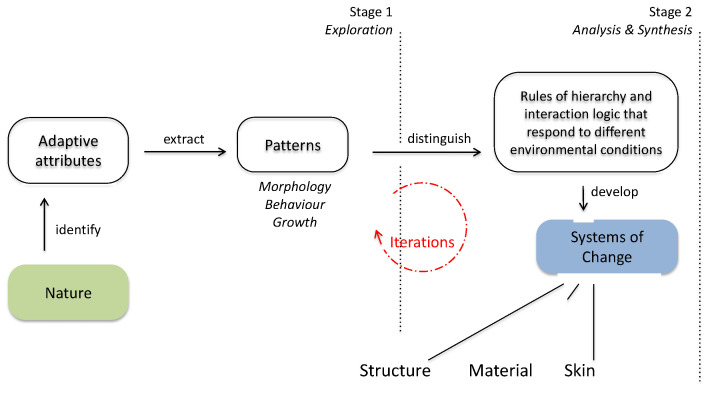
Diagram of two general stages involving an iterative design process for constructive learning. This diagram supports a design brief that aims to develop an understanding of how biology can be a model for adaptation, exploring the ways in which patterns of morphology, behavior, or growth can inform the development of architecture of change through structural, material, or skin systems.

Workshops and design studios further enhance experiential learning. For example, in the “Breathing Skins” workshop [94], students developed adaptive façades inspired by plant transpiration. Other classroom projects have included biomimetic shading devices modeled on termite mounds and self-cleaning cladding inspired by lotus leaves [95]. These projects combine observation, reflection, conceptualization, and prototyping. Templates and structured frameworks (e.g., Figure 9) guide students in defining and refining their ideas, enabling both independent inquiry and cooperative learning [96,97].

**Figure 10:**
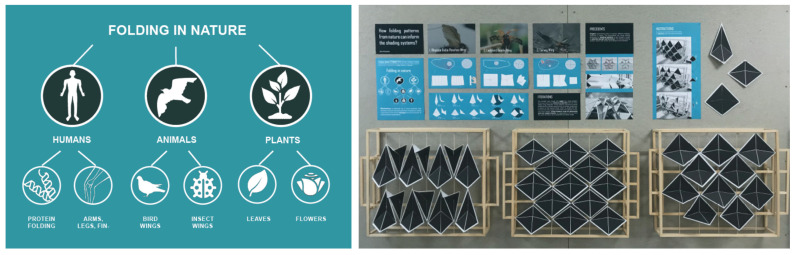
An adaptive shading system inspired by folding mechanisms from nature. Used with permission; image courtesy of Agne Arlauskaite, Design Research 2019/20.

**Figure 11:**
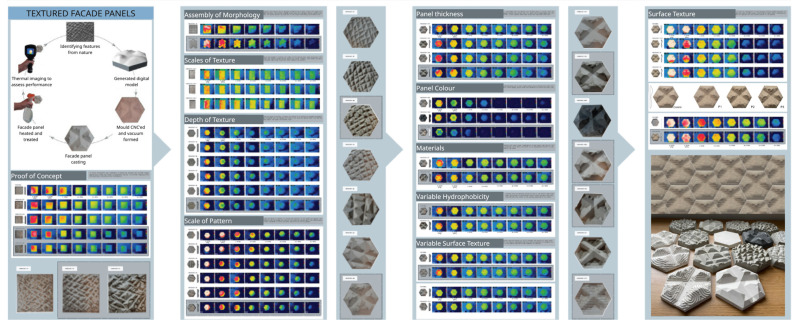
Systematic iterative design process for the development of textured façade panel inspired by elephant skins for evaporative cooling of buildings. Used with permission; image courtesy of Megan Peeks, Design Research 2019/20.

Hands-on experimentation is often complemented by reflective practice. For instance, students may examine how certain plant morphologies adapt to changing light levels and, through iterative prototyping (e.g., Figure 10), develop solar-responsive building systems. A project inspired by elephant skin produced a cooling façade panel that demonstrates how surface texture influences thermal performance [98], the systematic iterations are presented in Figure 11 through identifying relevant variables for validation. This mirrors biological adaptation through trial and error, emphasizing learning as a cyclical process of testing, feedback, and optimization.

Together, constructivist and experiential approaches position learners as active agents in the creation of knowledge. They foster critical thinking, creativity, adaptability, and collaboration—skills necessary to navigate interdisciplinary challenges and real-world sustainability goals. As Leal Filho et al. [99] argue, a quality education for sustainable development involves learning environments that support values-based inquiry, systems awareness, and collaborative engagement. By embedding these approaches into education, students are empowered to not only absorb knowledge but also challenge assumptions, iterate solutions, and design for resilience in a rapidly evolving world.

## 7. Integrated Curriculum Development

**Figure 12:**
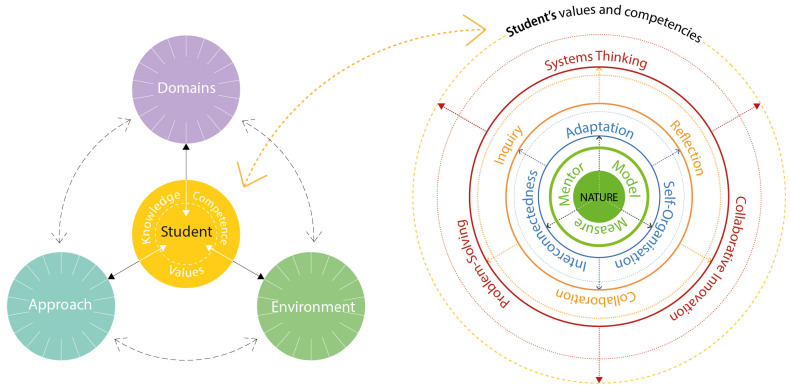
The integrated curriculum model. (**Left**) Diagram illustrating the dynamic interplay between the student, subject domains, pedagogic approaches, and the learning environment. (**Right**) As we zoom in (dashed yellow arrow), a multi-layered student-centric biomimetic pedagogic model is presented, demonstrating the four nested dimensions that shape their values and competencies: the central hub (green) represents the foundation of nature as a model, measure, and mentor; the central hub informs the core principles (blue) of adaptation, interconnectedness, and self-organization; the pedagogic processes (orange) based on reflection, inquiry, and collaboration encircle the core principles; theory and practice are bridged through transformative competencies (red) of systems thinking, problem-solving, and collaborative innovation. All of these contribute to a cohesive and integrated learning experience for students.

An integrated curriculum emphasizes interconnectedness, breaking down traditional disciplinary boundaries to create a cohesive and holistic learning experience. This approach aligns closely with the principles of biomimetic education, where collaboration, problem-solving, and systems thinking are central. In developing an integrated curriculum, four key areas—*students*, *learning and teaching environment*, *pedagogic approaches*, and *subject domains*—interact dynamically to shape a cohesive and transformative educational framework, as presented in Figure 12 (left). The student, as an active participant, embeds values, develops competencies, and acquires knowledge through reciprocal interactions with these elements, fostering a holistic and systems-oriented educational experience, as presented in Table 2. The teacher has a significant role in facilitating this interaction, where an integrated approach can be enriched through research-led and problem-based paradigms encountering aspects of materials, technical details, construction processes, design concepts, environment, and creativity [100,101]. In this context, the interdisciplinary and multifaceted nature of biomimetics offers significant potential when meaningfully integrated into education, enabling a transformative model grounded in nature’s core principles—adaptation, interconnectedness, and self-organization—for deeper understanding, creativity, and systems thinking, as illustrated in Figure 12 (right). This illustration builds on the key aspects identified from relevant theories and examples discussed in Section 3, Section 4, Section 5 and Section 6, which emphasize inquiry-based learning, interdisciplinary collaboration, and reflective practices to foster critical thinking, creativity, and sustainability-focused problem-solving among students. Central to this model is the concept of learning from nature to address real-world challenges, where natural systems serve as a model, measure, and mentor, emphasizing the profound lessons that natural systems offer in terms of resilience, adaptability, and sustainability [20].

### 7.1. The Integrated Curriculum Model

At the heart of the curriculum are the *students*, who are envisioned as active participants in their learning journey. Rather than passively receiving information, they engage in inquiry, critical thinking, and problem-solving, becoming co-creators of knowledge. The curriculum focuses on fostering values, such as ecological empathy, ethical responsibility, and sustainability, alongside competencies like systems thinking, collaborative innovation, and adaptability. Students also acquire knowledge to develop interdisciplinary understandings, connecting principles from biology, design, engineering, and environmental science to develop practical, sustainable solutions. Combining knowledge and competence is challenging, where there is a need to balance between input (knowledge) and output (competence)-oriented thinking [7].

The *learning and teaching environment* plays a crucial role in fostering inclusivity, collaboration, and adaptability. By offering flexible spaces and tools, it accommodates diverse needs and emphasizes the value of embracing differences. This approach ensures that all students feel supported and empowered to engage meaningfully in their learning journey, while also providing varied opportunities for growth and exploration [102,103,104,105]. Real-world contexts, such as field trips, community-based projects, and design and fabrication labs, bridge theoretical learning with practical application [82]. In this environment, students not only learn from their surroundings but also shape the learning experience through active participation and feedback, creating a shared sense of ownership. Joore, et al. [106] emphasize the role of living labs learning environments in fostering hands-on, collaborative, and interdisciplinary learning, where students and researchers co-create solutions to real-world challenges. Living labs, as described, provide a dynamic context for biomimetics education by enabling iterative experimentation and reflection.

*Pedagogic approaches* form the foundation for how the curriculum is delivered. Integrated curricula leverage inquiry or project-based learning (PBL) to provide students with hands-on opportunities to apply their knowledge. It encourages students to ask questions and explore natural systems for solutions, fostering curiosity and creativity. Experiential learning emphasizes hands-on projects and real-world problem-solving, ensuring that theoretical knowledge is connected to tangible outcomes. Reflective practices guide students to critically assess their decisions and iterate their solutions, fostering adaptability, resilience, and a deeper understanding of their work’s ethical and social implications.

Finally, the *subject domains* provide the content and thematic focus of the curriculum. These domains are interconnected, reflecting the interdisciplinary nature of real-world challenges. Central themes, such as social justice and equity [107], climate resilience, or regenerative design, unify the curriculum, enabling students to explore topics like resource efficiency or biodiversity conservation from multiple perspectives. This thematic approach ensures relevance by demonstrating how knowledge from diverse domains converges to address global challenges holistically, equipping students with the tools to develop integrated solutions [49,108].

### 7.2. Student-Centric Biomimetic Pedagogic Model

The model is structured across three interrelated layers grounded in student learning experience. At the innermost layer are the core principles of adaptation, interconnectedness, and self-organization. Adaptation emphasizes the importance of flexibility and responsiveness to changing conditions, mirroring nature’s strategies for survival. This principle encourages students to embrace uncertainty and refine their solutions iteratively. Interconnectedness highlights the synergies and relationships within ecosystems, fostering systems thinking and a holistic understanding of complex problems. Self-organization, inspired by emergent phenomena in nature, promotes student autonomy and collaboration, enabling learners to manage their own goals and co-create innovative solutions.

The middle layer focuses on the pedagogical processes that operationalize these principles. Inquiry forms the foundation of the learning experience, encouraging students to observe natural systems, ask critical questions, and derive design principles from their observations. For instance, students might study how trees manage water distribution and apply these insights to urban water infrastructure projects. Collaboration integrates interdisciplinary perspectives, bringing together knowledge from biology, engineering, design, and social sciences to tackle multifaceted challenges. Reflection ensures that students critically evaluate their decisions and refine their approaches, aligning with Schon [63] model of reflective practice. This iterative process fosters resilience, adaptability, and deeper learning.

At the outermost layer, the model defines the learner values and competencies that emerge from biomimetic education. These include systems thinking, which enables students to understand and address interconnected problems holistically, and problem-solving, which focuses on designing actionable, sustainable solutions. Collaborative innovation is also a central outcome, emphasizing the ability to work across disciplines to co-create impactful ideas. These competencies not only prepare students to tackle real-world challenges but also instill a commitment to sustainability, creativity, and ecological stewardship.

## 8. Towards a Holistic Educational Framework

This section presents a novel integrated, multidimensional pedagogic framework designed to support a holistic, systems-oriented approach to education. It builds on the growing awareness that teaching and learning must be approached from a cognitive perspective, acknowledging that while subject domains, environments, educators, and learners evolve over time, the learning process itself follows cognitive patterns that shape knowledge acquisition and competency development [109,110]. Understanding these patterns enhances the capacity of educators to adapt teaching strategies to diverse learning contexts, creating stronger cognitive connections and fostering more effective, transferable learning experiences [109,111,112,113]. This perspective aligns with current calls for system-wide educational transformation that supports complex, real-world problem solving and interdisciplinary engagement [114].

In this context, shared meaning becomes a foundational component of holistic, systems-oriented education. Establishing common conceptual ground—around themes such as adaptation, resilience, and sustainability—not only supports interdisciplinary dialogue but also cultivates collective responsibility and collaborative innovation. It enables students from diverse backgrounds to connect knowledge across domains and co-create solutions informed by integrated perspectives. This process reflects the interconnected nature of ecological systems and mirrors the collaborative dynamics required in real-world design contexts.

**Figure 13:**
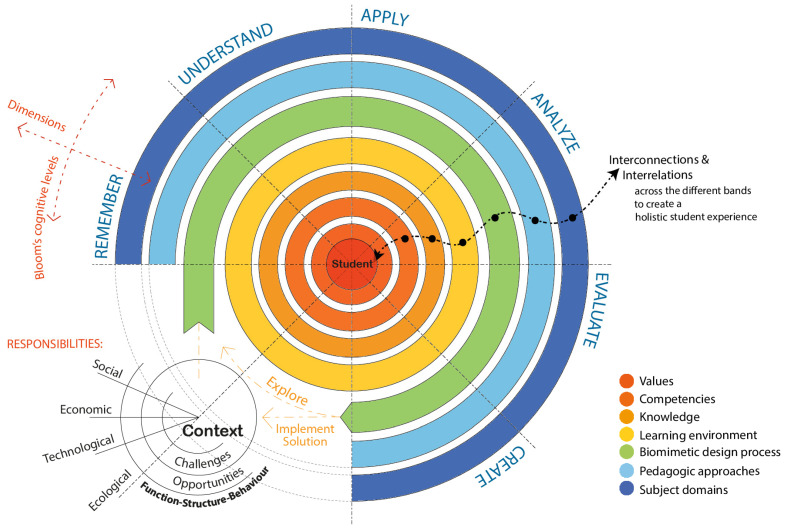
A holistic approach to integrated curriculum design, using Bloom’s Taxonomy as a cognitive scaffold and context (e.g., social, economic, technological, ecological) as responsibility drivers, while embedding biomimetics as nature-inspired thinking to problem-solving. In a student-centric educational system, the nested dimensions of *values*, *competencies*, *knowledge*, *learning environment*, *biomimetics*, *pedagogic approaches*, and *subject domains* are aligned across the cognitive levels to facilitate interconnections and interrelations through Function–Structure–Behavior (FSB) schema and develop the relevant competencies.

To scaffold this approach cognitively, the framework draws on Bloom’s Revised Taxonomy [26] as a structure for organizing learning progression—from remembering and understanding to applying, analyzing, evaluating, and creating. Within this scaffold, biomimetics functions as the primary methodology for innovation and problem-solving. This dual structure—Bloom’s Taxonomy as the cognitive framework and biomimicry as the design methodology—enables learners to move systematically from a conceptual understanding of natural systems to the synthesis of regenerative, adaptive solutions in the built environment. Figure 13 illustrates this student-centered, integrative framework that aligns learning progression with Bloom’s Taxonomy, moving from foundational stages such as Remember and Understand toward higher-order thinking skills like Create. It visually maps the layered dimensions of learning progression in a biomimetic integrated educational model, with each concentric band representing a crucial distinct component yet interconnected, contributing to holistic development.

At the center lies the *student*, emphasizing personal growth, engagement, and transformation as the aim of the learning journey. The student is directly linked with the contextual layers, which include ecological, technological, economic, and social factors. Context defines the types of challenges and opportunities students are expected to address and engage with, guiding the selection of relevant competencies and subject domains. The Function–Structure–Behavior (FSB) schema is applied holistically—from the starting point of context—to guide both analysis and synthesis across all dimensions of curriculum design and delivery. Context frames the initial function: it defines the purpose, situational drivers, or real-world challenges that necessitate learning. This contextual understanding sets in motion the identification of appropriate structures and anticipates expected behaviors or outcomes.

Surrounding the student is the *values* band (deep red), which underpins the entire framework. These values—such as curiosity, responsibility, empathy, and ethical awareness—form the internal compass guiding students’ decisions and actions. They are essential in shaping not only what students learn but also why and how they apply their knowledge, particularly in response to complex societal and environmental challenges. Next is the *competencies* band (orange), which captures transferable, cross-disciplinary abilities such as critical thinking, collaboration, systems thinking, adaptability, and creativity. These competencies are cultivated as students move outward through the cognitive stages of Bloom’s Taxonomy and are crucial for enabling learners to engage with complexity and uncertainty. They are both informed by and applied to the contextual challenges identified in the surrounding environment.

The *knowledge* band (light orange) represents the foundational and domain-specific understanding that supports competency development. It includes both theoretical and applied knowledge, derived from multiple disciplines. The selection and depth of knowledge are context-dependent, shaped by real-world issues and the desired learning outcomes. Encircling this is the *learning environment* band (yellow), which encompasses the physical, digital, and social conditions in which learning occurs. It includes studio-based settings, fieldwork, co-creation workshops, and digital platforms that support active, collaborative, and iterative learning. A responsive learning environment allows students to explore, reflect, and test ideas in diverse contexts, reinforcing knowledge and skill development through experiential engagement.

The *biomimetic design process* band (green) brings methodological structure to problem-solving. This process is informed by nature’s strategies and structured around the FSB schema. It guides students to first understand the functions needed to address a challenge, explore how biological systems achieve these functions (structure), and examine the behaviors that emerge in context. This fosters a shift from linear problem-solving to systems-based, adaptive thinking.

The *pedagogic approaches* band (light blue) provides instructional methods aligned with different stages of cognitive development. It includes scaffolded learning in the early stages (e.g., guided discovery, storytelling), and progressively opens into methods that support synthesis and innovation (e.g., design-based learning, collaborative projects, transdisciplinary exploration). The choice of pedagogy supports disciplinary aims and cognitive and affective development, reinforcing alignment across the framework.

Finally, the *subject domains* band (outer dark blue) reflects the breadth and adaptability of content areas. These domains are selected in response to contextual imperatives—social, ecological, technological, and economic—as identified in the context circle. Each domain addresses challenges and opportunities by drawing on relevant knowledge and methodologies. The flexibility of this band allows curriculum to remain dynamic and responsive to current and emerging global needs.

Together, these bands operate as an interconnected system that links inner development (values, competencies) with external engagement (subject domains, context). The framework encourages students to move through different cognitive levels, navigate across dimensions, engage with interdisciplinary content, integrate learning through relevant pedagogic strategies, develop design responses informed by biomimetic design processes, and become agents of informed, responsible innovation. Moreover, the FSB schema functions as a transversal analytical and generative tool, enabling systemic alignment and knowledge transfer across the curriculum. It brings coherence and integrative thinking to complex educational settings, particularly in fields like architecture, design, engineering, and other interdisciplinary domains. Overall, the framework emphasizes the importance of embedding competencies in a learning journey that is both cognitively rigorous and contextually grounded. Figure 13 and Table 3 conceptually illustrate this alignment and interconnectedness.

Through this integration, students engage in a learning process that supports both intellectual development and practical creativity. For instance, a learner might begin by studying the thermal regulation of termite mounds (understanding), investigate how this is achieved structurally and behaviorally (analyzing), and then apply this insight to design a passive cooling system for a building (creating). This progression fosters a deeper grasp of systems thinking, ecological literacy, and interdisciplinary synthesis.

The proposed biomimetic pedagogic framework thus embodies a transformative educational model—one that equips students with not only the knowledge and skills but also the values required to address complex global challenges. Its layered design ensures a holistic development of learners, where cognitive growth is accompanied by the cultivation of systems thinking, ethical reasoning, and collaborative competence.

By embedding inquiry, collaboration, and reflection at its core, the framework supports experiential, constructivist learning processes that mirror natural evolution—adaptive, iterative, and emergent. It enables students to move beyond traditional disciplinary silos and develop the capacity to design resilient, regenerative systems that respond to the pressing demands of sustainability and climate adaptation.

Ultimately, this nature-inspired, cognitively structured approach contributes to the development of a new educational paradigm—one that is purpose-driven, adaptive, and capable of preparing learners to shape more sustainable and equitable futures.

## 9. Discussion

The novel holistic biomimetic pedagogic framework presented in this study offers a transformative approach to architectural education, sustainability, and innovation. By aligning pedagogical practices with nature’s principles—adaptation, interconnectedness, and self-organization—the framework cultivates a generation of learners equipped to address complex global challenges through systems-oriented, ethical, and creative approaches. This reflects the values embedded in Benyus [20] biomimicry lens and supports the development of key competencies for sustainability such as systems thinking, collaboration, and ecological literacy [50,51,52].

A central impact of this framework lies in its integration of systems thinking as a foundational competency. Drawing from Capra’s [5] and Ison’s [15] systems paradigm, the framework encourages students to recognize interdependencies and feedback loops, which are essential for addressing wicked challenges like climate change and resource scarcity. In educational practice, this systems perspective is effectively modeled through the Function–Structure–Behavior (FSB) schema [8,10,36,70]. For example, students exploring thermoregulation in termite mounds learn to analyze the function of temperature control, examine the structure of ventilation systems, and simulate behavior under different environmental conditions. This triadic analysis enables learners to move beyond surface-level analogies toward a deeper abstraction of biological principles for architectural application [40,73].

The FSB approach, along with a structured template, has been successfully embedded in studio-based and workshop settings. For instance, in the Design Research module, students developed foldable shading devices for facade systems inspired by plants and insect wings, illustrating how observing and abstracting behavior from nature supports the creation of adaptive architectural skins. Similarly, the Material Incubator project at UWE Bristol applied biomimetic thinking to endemic materials and local ecologies, using systems thinking to define FSB relationships in their design.

To facilitate progression from novice to expert thinking, the framework aligns with Bloom’s Revised Taxonomy [26] as a cognitive scaffold. Students move from remembering biological strategies and understanding their ecological contexts to applying them in abstracted design challenges, analyzing structure–function–behavior interactions, evaluating the performance and sustainability of their solutions, and ultimately creating novel, regenerative architectural systems. Mapping this progression enables the systematic development of cognitive, technical, and ethical capacities in students [24,30].

The framework is operationalized through inquiry-led, hands-on pedagogies that foster constructivist and experiential learning [17,63]. As evidenced in Studio One [82] and design research projects at UWE Bristol, students engage in iterative experimentation, prototyping, and critique—processes that mirror natural evolution. Concept maps, FSB diagrams, and systems models are employed to externalize thinking and strengthen interdisciplinary synthesis [76,77,78]. These methods help students visualize and navigate the complexity of biological and built systems. The framework’s emphasis on interdisciplinarity is further reflected in courses where architects collaborate with biologists, engineers, and environmental scientists. Living labs and community-based projects exemplify how transdisciplinary learning environments support ecological empathy and co-creation [106]. As Jacobs, Eggermont, Helms, and Wanieck [14] and Wanieck, Ritzinger, Zollfrank, and Jacobs [16] emphasize, such settings build cognitive flexibility and creative problem-solving capacity—essential traits for designing within socio-ecological systems. Importantly, this biomimetic approach reshapes the ethical orientation of architectural education. By positioning nature as mentor rather than resource, learners cultivate ecological empathy and stewardship [12,20,55]. The visit to the University of Bristol Botanic Garden illustrates how field-based inquiry enhances students’ ability to observe, reflect, and abstract design principles while fostering a deeper relational understanding of nature.

The implications of this framework also extend to the institutional and policy levels. Embedding biomimetic principles requires educational systems to adopt experiential, inquiry-based, and transdisciplinary curricula [2,13,50]. Breaking down disciplinary silos and reframing design briefs to address real-world ecological challenges—as advocated by McCormick and Thaddeus [41] and Pedersen Zari [43]—positions students to act as agents of regenerative transformation in the built environment.

In summary, the student-centric framework bridges Bloom’s cognitive taxonomy with biomimicry’s innovation capacity and systems thinking’s function–structure–behavior schema to support a holistic multi-layered learning process. It enables students to develop not only technical design capabilities but also the critical, reflective, and systems-oriented mindsets needed to navigate complexity. When situated in hands-on, studio-based, and interdisciplinary contexts, this framework fosters the development of resilient, responsible designers capable of shaping sustainable and regenerative futures.

## 10. Conclusions

Biomimetics offers a transformative approach to education by integrating innovation, sustainability, and systems thinking into pedagogical practice. Grounded in nature’s principles of adaptation, interconnectedness, and self-organization, the proposed integrated framework cultivates critical thinking, creativity, and the capacity to develop holistic, systems-oriented solutions. By embedding systems thinking within biomimicry education, students gain the tools and adaptive mindset needed to address complex, interdependent challenges. This approach not only enhances problem-solving abilities but also aligns design strategies with ecological resilience and ethical responsibility.

The framework emphasizes constructivist and experiential learning, encouraging students to actively engage with nature through observation, reflection, and application. It supports interdisciplinary collaboration and promotes real-world, project-based learning environments where students prototype and iterate design concepts inspired by biological systems. Such contexts help foster ecological empathy, collaborative innovation, and systems awareness. Importantly, the integration of Bloom’s Revised Taxonomy with the Function–Structure–Behavior (FSB) schema provides a clear cognitive and methodological scaffold. This alignment supports the progression from foundational knowledge to advanced application and innovation, guiding students from understanding natural systems to designing regenerative architectural solutions. Studio-based courses and living labs demonstrate how these principles translate into practice, empowering learners to explore complex systems and apply biomimetic insights with creativity and rigor.

Ultimately, this framework repositions education as a catalyst for systemic transformation. By learning with and from nature, students are equipped to co-create sustainable solutions that reflect ecological principles and societal needs. In doing so, biomimetic education not only redefines how future architects and designers learn—it lays the groundwork for a regenerative and resilient future, where learning becomes an emergent process for all.

## Figures and Tables

**Table 1:** The structure of Bloom’s Revised Taxonomy on the cognitive process dimensions, adapted from Krathwohl [26].

Dimension	Examples of Competencies	Definition
Remember	Recognizing; Recalling	Retrieving relevant knowledge from long-term memory
Understand	Interpreting; Exemplifying; Classifying; Summarizing; Inferring; Comparing; Explaining	Determining the meaning of instructional messages, including oral, written, and graphic communication.
Apply	Executing; Implementing	Carrying out or using a procedure in a given situation.
Analyze	Differentiating; Organizing; Attributing	Breaking material into its constituent parts and detecting how the parts relate to one another and to an overall structure or purpose.
Evaluate	Checking; Critiquing	Making judgments based on criteria and standards.
Create	Generating; Planning; Producing	Putting elements together to form a novel, coherent whole or make an original product.

**Table 2:** The core elements of integrated curriculum development. The key areas of the integrated curriculum highlight how values, skills, and knowledge interact to foster holistic and systems-oriented learning in biomimetic education.

	Values	Competencies	Knowledge
Students	Ecological empathy, ethical responsibility, and sustainability	Systems thinking, problem-solving, collaborative innovation	Interdisciplinary understanding of biomimicry, sustainability, and natural principles
Learning and Teaching Environment	Inclusivity, respect, and shared ownership of the learning process	Collaboration, adaptability, and engagement in real-world contexts	Application of theoretical knowledge to practical settings like fieldwork and design labs
Pedagogic Approaches	Reflection on ethical and social responsibilities	Critical thinking, inquiry, and iterative design processes	Thematic, interdisciplinary integration of disciplines such as biology, design, and engineering
Subject Domains	Accountability and stewardship through addressing global challenges like climate resilience	Application of systems thinking and interdisciplinary methods to solve complex problems	Knowledge of interconnected systems and principles like adaptation, resilience, and circularity

**Table 3:** Examples of aspects for the Bloom’s Taxonomy curriculum mapping matrix.

Bloom’s Taxonomy	Remember	Understand	Apply	Analyze	Evaluate	Create
**Biomimetics**	Explore biological knowledge	Interpret natural functions	Abstract strategies and translate into solutions	Experiment and analyze relationships	Assess the appropriateness of the solution	Develop prototypes
**Subject domain**	e.g., Biological foundation	e.g., Ecological systems	Biological strategies in design	Cross-domain systems thinking	Performance and sustainability evaluation	Transdisciplinary innovation
**Pedagogical approach**	Direct instructions	Concept mapping, analogical reasoning	Inquiry-based learning	Systems mapping and critique	Reflective practice	Project-based and experiential learning
**Environment**	Seminar rooms, digital archives	Studio, lab, field observations	Studio, structured design briefs	Workshops, collaborative studios	Labs, critique panels, stakeholder engagement spaces	Living labs, co-creation spaces
**Student role**	Recall facts	Explainer, relationship builder	Practitioner, problem-solver	Analytical thinker	Critical evaluator	Maker, innovator
**Values**	Appreciation for nature’s complexity and adaptation	Curiosity and openness	Responsibility and action orientation	Systems awareness and transparency	Ethical reasoning and sustainability	Innovation and ecological empathy
**Knowledge**	Factual and conceptual	Interpretive and contextual	Procedural and applied	Relational and comparative	Ethical and evaluative	Integrated and fit for purpose
**Competencies**	Identification and classification	Explanation and synthesis	Application and translation	Analysis and abstraction	Evaluation and justification	Creative synthesis and prototyping

## Data Availability

Data is contained within the article.
